# Barriers to Diagnosis and Treatment of *Clostridioides difficile* Infection: A Narrative Review

**DOI:** 10.3390/antibiotics15101004

**Published:** 2026-10-10

**Authors:** Parul Berry, Vishwesh Bharadiya, Sahil Khanna

**Affiliations:** 1 Department of Internal Medicine, Mayo Clinic, Rochester, MN 55905, USA; berry.parul@mayo.edu (P.B.); bharadiya.vishwesh@mayo.edu (V.B.); 2 Division of Gastroenterology and Hepatology, Mayo Clinic, Rochester, MN 55905, USA

**Keywords:** *Clostridioides difficile* infection, recurrent CDI, diagnostic stewardship, fidaxomicin, vancomycin, fecal microbiota transplantation, VOWST, REBYOTA, microbiota-based therapies

## Abstract

*Clostridioides difficile* infection (CDI) remains a major cause of healthcare-associated diarrhea and is being increasingly recognized in community settings. The management of CDI is complicated by diagnostic uncertainty, recurrent disease, and uneven access to newer therapies. Clinicians must distinguish infection from colonization, individualize therapy selection, and anticipate barriers to recurrence prevention. This narrative review examines diagnostic and therapeutic barriers in adult CDI care, with particular emphasis on the U.S. healthcare, regulatory, and reimbursement setting. Several guidelines prefer fidaxomicin for an initial nonfulminant CDI episode when available. Oral vancomycin is an effective alternative, and metronidazole is reserved for initial nonsevere CDI when preferred agents are unavailable. Antibiotic options for recurrent CDI include standard or extended-pulsed fidaxomicin and standard-course or tapered-and-pulsed vancomycin regimens. After antibacterial treatment, conventional fecal microbiota transplantation (FMT) and two FDA-approved microbiota products, fecal microbiota spores, live-brpk (VOS) and fecal microbiota, live-jslm (RBL), may be used in appropriately selected patients to prevent another recurrence. In ECOSPOR III, recurrence through 8 weeks was 12.4% of VOS recipients and 39.8% of placebo recipients. In PUNCH CD3, Bayesian model–estimated treatment success through 8 weeks was 70.6% with RBL and 57.5% with placebo. Because the trials used different populations, endpoints, definitions, and analytical frameworks, these figures should not be interpreted as a head-to-head comparison. The discontinuation of bezlotoxumab in the United States and reduced availability of stool bank-derived FMT have further constrained recurrence prevention options. Practical considerations include diagnostic stewardship, assessment of recurrence risk, and timely access to indicated recurrence prevention therapy.

## 1. Introduction

*Clostridioides difficile* infection (CDI) remains a leading cause of healthcare-associated diarrhea in the United States, affecting approximately half a million individuals with an estimated 12,000–30,000 attributable deaths annually [1,2]. Annual per-patient direct medical costs associated with recurrent CDI (rCDI) are estimated at $67,000 to $82,000 [3]. Although healthcare-associated CDI incidence has declined, community-associated CDI remains an important component of U.S. disease burden [1]. Up to 35% of patients experience a recurrence after an initial CDI, and up to 60% of patients with a first recurrence experience additional recurrences [4,5,6].

Internists, hospitalists, and primary care clinicians are central to CDI care because they often make the initial diagnostic and therapeutic decisions [7]. Accurate diagnosis is challenging because asymptomatic colonization is common and nucleic acid amplification tests (NAATs) detect toxigenic genes but do not establish active toxin production [8].

Fidaxomicin is the guideline-preferred therapy for first CDI episode when feasible, but cost and insurance restrictions can limit access [9]. Oral vancomycin (125 mg four times daily for 10 days) remains widely used, whereas metronidazole now has a limited role in selected low-risk cases because of lower efficacy and higher recurrence rates [10,11]. Two FDA-approved donor-derived microbiota restoration therapies, fecal microbiota, live-jslm (REBYOTA; abbreviated as RBL for brevity), and fecal microbiota spores, live-brpk (VOWST; abbreviated as VOS for brevity), are now available for recurrence prevention after antibacterial treatment [12,13,14,15]. At the same time, the U.S. discontinuation of bezlotoxumab and reduced access to conventional stool bank-derived FMT have constrained recurrence prevention options available [16,17].

A recent survey found that fewer than 10% of physicians considered themselves very knowledgeable about donor-derived microbiome therapies [18]. This review examines diagnostic uncertainty, antibiotic selection, recurrence prevention, and barriers to accessing recommended therapies in adult CDI care. It focuses on how guideline recommendations are implemented in U.S. practice by integrating diagnostic stewardship, medication coverage, product-ordering logistics, FMT regulatory changes, and care transitions.

### Review Approach

The literature was searched in PubMed and MEDLINE from January 2000 through August 2026 using combinations of the terms “*Clostridioides difficile*,” “*C. difficile*,” diagnosis, diagnostic stewardship, fidaxomicin, vancomycin, fulminant infection, recurrence, fecal microbiota transplantation, microbiota-based therapy, access, insurance, prior authorization, and reimbursement. Current adult CDI guidance from the Infectious Diseases Society of America and Society for Healthcare Epidemiology of America (IDSA/SHEA), the European Society of Clinical Microbiology and Infectious Diseases (ESCMID), American College of Gastroenterology (ACG), and American Gastroenterological Association (AGA) was prioritized, together with pivotal randomized trials, systematic reviews, and official U.S. regulatory, payer, and manufacturer materials relevant to treatment access. Because this was a narrative review, study selection was not conducted under a registered systematic-review protocol and no formal risk-of-bias assessment was performed. The search was restricted to English-language publications involving adults. No restrictions were applied by publication type.

## 2. Evidence-Based Guidelines: Current Landscape

Major guidelines from ESCMID and IDSA/SHEA prefer fidaxomicin for an initial CDI episode when feasible, with oral vancomycin as an acceptable alternative. The ACG recommends either oral vancomycin or fidaxomicin for initial nonfulminant CDI. Metronidazole has a limited role in selected patients with initial nonsevere CDI [19,20,21].

For first recurrence, IDSA/SHEA conditionally suggests standard or extended-pulsed fidaxomicin over a standard course of vancomycin. A standard course of vancomycin or a tapered and pulsed vancomycin regimen remains an acceptable alternative [4,19]. For multiple recurrences, options include standard or extended-pulsed fidaxomicin, tapered and pulsed vancomycin, vancomycin followed by rifaximin, and FMT after antibiotics [22].

The AGA 2024 guideline addresses fecal microbiota-based therapies, including the FDA-approved donor-derived microbiota-restoration therapies, VOS and RBL, for rCDI prevention. In immunocompetent adults with rCDI, AGA suggests fecal microbiota-based therapy after standard-of-care antibiotics for rCDI prevention. In mildly or moderately immunocompromised adults with rCDI, AGA conditionally suggests conventional FMT. In severely immunocompromised adults, AGA conditionally recommends against fecal microbiota–based therapies. For selected adults hospitalized with severe or fulminant CDI that has not responded to standard antimicrobial treatment, AGA conditionally suggests conventional FMT as an adjunct [19,22,23,24,25].

Recommendations against conventional FMT in ulcerative colitis, Crohn disease, pouchitis, and irritable bowel syndrome (IBS) refer to FMT as treatment for those underlying disorders outside clinical trials. They do not preclude use for recurrent, severe, or fulminant CDI in a patient with one of these conditions.

## 3. Clinical Barriers

### 3.1. Diagnostic Challenges

**Figure 1:**
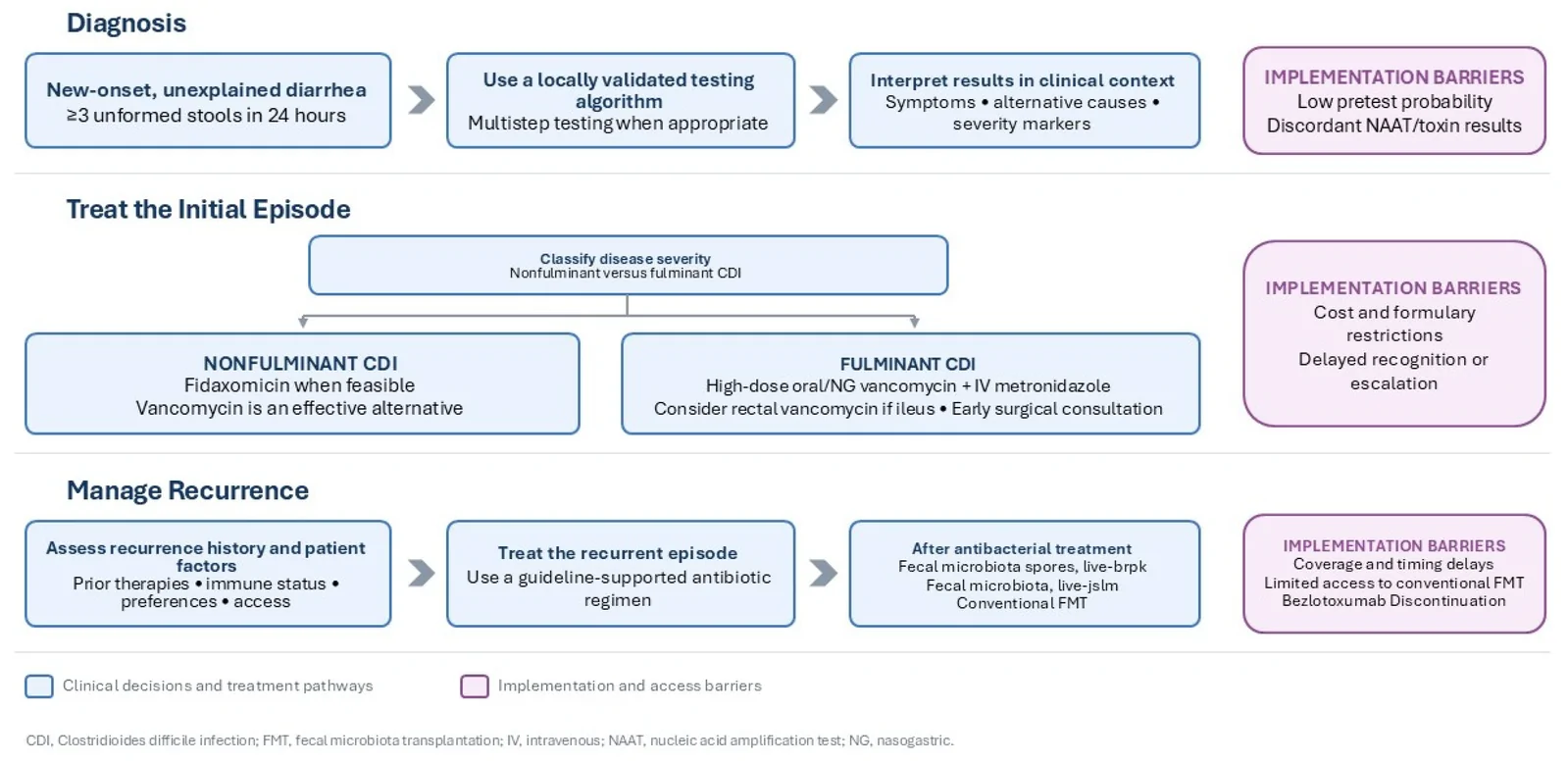
Diagnostic and treatment pathway for adult CDI in U.S. practice.

Accurate and timely diagnosis remains a major barrier to appropriate CDI treatment [26]. The diagnostic approach is summarized in Figure 1. Symptoms such as diarrhea, abdominal pain, and fever are non-specific and can overlap with inflammatory bowel disease (IBD), tube feeding, laxative exposure, medication-related diarrhea, and IBS [27]. Diagnosis should therefore integrate symptoms, risk factors, and local laboratory testing protocols.

Common diagnostic tests include NAATs for toxigenic *C. difficile* genes, toxin enzyme immunoassays (EIAs), glutamate dehydrogenase (GDH) EIAs, and, less commonly, toxigenic culture or cell cytotoxicity assays. NAATs are highly sensitive but cannot distinguish colonization from toxin-mediated disease. Toxin EIAs detect active toxin in stool and are more specific but are less sensitive [4,28]. This tradeoff creates a common dilemma: NAAT-positive/toxin-negative results.

NAAT-positive/toxin-negative patients often have lower markers of disease severity than toxin-positive patients, suggesting that many represent colonization or milder disease. In a 2024 meta-analysis of observational studies, treated NAAT-positive/toxin-negative patients had lower 30-day all-cause mortality than untreated patients, implying that some toxin-negative patients have clinically meaningful CDI. However, the authors noted that confounding and selection bias limited causal interpretation [29,30]. Therefore, discordant results should not automatically trigger or preclude therapy. Treatment decisions should incorporate the clinical presentation, alternative causes of diarrhea, leukocytosis, imaging findings, and evidence of severe or fulminant disease.

### 3.2. Multistep Testing and Diagnostic Stewardship

To balance sensitivity and specificity, many institutions have adopted multistep diagnostic algorithms. The 2017 IDSA/SHEA guideline recommends including a stool toxin test in a multistep algorithm when institutions do not have agreed-upon criteria for stool submission. One approach uses GDH and toxin EIAs, with NAAT to resolve discordant results. In practice, testing protocols vary widely [31]. In a College of American Pathologists survey, 59.0% of 1159 responding laboratories used multistep algorithms, while others used NAAT alone or NAAT followed by toxin testing. The laboratories also varied regarding formed-stool rejection and testing after laxative exposure.

Diagnostic stewardship aims to restrict testing to patients with a reasonable pretest probability of CDI. Testing should generally be limited to patients with new-onset, unexplained diarrhea and should not be repeated within 7 days during the same diarrheal episode or used as a test of cure [32]. Electronic ordering prompts may require clinicians to document the number of unformed stools and recent laxative exposure before placing an order. Other interventions include laboratory rejection of formed stool, cancelation of duplicate tests, audit and feedback, and clinician education. These interventions reduce testing in patients unlikely to have CDI and may therefore reduce false-positive diagnoses and unnecessary treatment [2,33,34].

### 3.3. Special Diagnostic Contexts

The usual target population for CDI testing is adults with at least 3 unformed stools in 24 h without another clear explanation [4]. Patients with suspected fulminant CDI may have ileus and little or no diarrhea. Abdominal distension, fever, leukocytosis, hypotension, or toxic megacolon should therefore prompt evaluation even when an unformed stool specimen cannot be obtained. At institutions whose laboratories have validated rectal swabs for molecular testing, a visibly stool-soiled rectal swab may be considered after consultation with the laboratory.

## 4. Therapeutic Controversies and Challenges

### 4.1. Initial CDI Therapy: Fidaxomicin vs. Vancomycin

Fidaxomicin has initial clinical cure rates comparable to vancomycin but is associated with a lower risk of recurrence, likely because its narrower spectrum causes less disruption of the intestinal microbiota [35]. The IDSA/SHEA suggests fidaxomicin over a standard course of vancomycin for an initial nonfulminant CDI episode while explicitly noting that implementation depends on available resources. When fidaxomicin is unavailable or not covered, oral vancomycin, 125 mg 4 times daily for 10 days, remains an effective alternative [19,21].

Incidental systemic antibiotics should be discontinued when possible, and antibiotics that must be continued should be narrowed to the shortest effective course. Acid-suppressive therapy should also be reassessed and continued only when clinically indicated [24]. Although clinically important resistance to fidaxomicin and vancomycin remains uncommon, surveillance studies have identified evolving antimicrobial resistance patterns in *C. difficile*, underscoring the need for continued stewardship [36,37]. Ibezapolstat is an investigational DNA polymerase IIIC (PolC) inhibitor with microbiome-sparing properties. It has completed phase 2 evaluation. Phase 3 evidence is needed before its role in CDI treatment can be established.

Clinical Application: An older adult with a first nonfulminant CDI episode, recent hospitalization, and ongoing systemic antibiotic exposure has an increased risk of recurrence. Fidaxomicin is preferred when it can be obtained without delaying treatment. When cost or formulary restrictions prevent timely access, oral vancomycin remains an effective guideline-supported alternative while pharmacy or case-management staff initiate coverage support.

### 4.2. Fulminant CDI

Severe CDI without hypotension, shock, ileus, or megacolon is treated with the same standard-dose fidaxomicin or oral vancomycin regimens used for nonsevere CDI. In contrast, fulminant CDI requires a different antibiotic regimen and urgent multidisciplinary management. Fulminant CDI is characterized by hypotension, shock, ileus, or megacolon and should prompt early surgical consultation and frequent reassessment for clinical deterioration. The IDSA/SHEA recommends vancomycin 500 mg four times daily orally or through a nasogastric tube with intravenous metronidazole. Rectal vancomycin may be considered when ileus limits drug delivery to the colon [24].

The AGA conditionally suggests conventional FMT as adjunctive therapy for selected hospitalized adults with severe or fulminant CDI that has not responded to antimicrobial treatment, although this recommendation is based on very-low-certainty evidence. Donor-derived microbiota restoration therapeutics, VOS and RBL, have not been studied as rescue therapies for fulminant CDI [22]. Use of RBL for fulminant CDI is off-label and should only be considered after multidisciplinary discussion of risks and benefits [38].

Illustrative clinical application. A hospitalized patient with CDI, hypotension, abdominal distension, and ileus should be managed as having fulminant disease. Treatment includes high-dose oral or nasogastric vancomycin plus intravenous metronidazole, consideration of rectal vancomycin when ileus limits colonic delivery, early surgical consultation, and frequent reassessment. Rescue conventional FMT is reserved for selected refractory cases after multidisciplinary review.

### 4.3. Recurrent CDI and Microbiome Restoration

Selection of therapy for recurrent CDI is guided by number of prior recurrences, previous therapies, disease severity, immunocompromised status, patient preference, and product access. Antibiotic options include standard or extended-pulsed fidaxomicin, a standard course or tapered-and-pulsed course of vancomycin, and, in selected multiply recurrent cases, vancomycin followed by rifaximin. After antibacterial treatment, conventional FMT, VOS, and RBL may be considered for recurrence prevention in appropriately selected patients [6,39,40].

Bezlotoxumab, a monoclonal antibody that binds *C. difficile* toxin B, reduced rCDI when added to standard antibacterial therapy in MODIFY I and II [41,42]. Although bezlotoxumab is no longer available in the United States, subgroup analyses illustrate the unmet need for recurrence prevention after a first high-risk CDI episode. Among participants without a CDI episode in the preceding 6 months, rCDI through 12 weeks occurred in 13.5% (75/556) of bezlotoxumab recipients and 20.9% (114/545) of placebo recipients. Among patients with at least one risk factor for recurrence after a primary episode, the corresponding rates were 16.3% (69/424) and 26.5% (106/400), an absolute difference of 10.1 percentage points (approximate number needed to treat, 10) [43].

IDSA/SHEA allowed consideration of bezlotoxumab during a primary episode in patients with additional recurrence risk factors or severe disease, whereas ESCMID generally positioned it within recurrence pathways [19,21,43]. Bezlotoxumab also carried a caution for patients with congestive heart failure. In the United States, Merck discontinued Zinplava in January 2025 and had been the sole supplier. No safety-related regulatory withdrawal was associated [17]. The European Medicines Agency continues to list a valid EU marketing authorization, although local commercial availability may vary [44]. RBL was FDA-approved in November 2022 as the first microbiota product for preventing rCDI. In PUNCH CD3, Bayesian model–estimated treatment success through 8 weeks was 70.6% with RBL and 57.5% with placebo [12]. VOS was FDA-approved in April 2023 as the first orally administered fecal microbiota product. In the ECOSPOR III trial, recurrence through 8 weeks occurred in 12.4% of VOS recipients compared with 39.8% of placebo recipients [13]. The trials differed in entry criteria, diagnostic definitions, and analytical frameworks. The percentages are therefore trial-specific and should not be interpreted as a head-to-head comparison (Table 1).

Neither trial directly compared VOS with RBL. Their comparative effectiveness, optimal sequencing, and effectiveness in populations underrepresented in trials remain uncertain. A pharmacovigilance analysis of 231 RBL and 813 VOS adverse event reports detected no disproportionality signals for bacteremia, septic shock, or anaphylaxis [45]. However, spontaneous-reporting data cannot establish event incidence or comparative safety.

Illustrative clinical application. In an adult completing antibiotics for a recurrent CDI episode, recurrence prevention planning should begin before the antibiotic course ends. Selection among conventional FMT, VOS, and RBL depends on immune status, route preference, local availability, payer approval, and the required post-antibiotic treatment window.

### 4.4. Special Populations

Immunocompromised patients require individualized assessment. After standard antibiotic treatment for rCDI, AGA conditionally suggests conventional FMT for mildly or moderately immunocompromised adults but suggests against all fecal microbiota–based therapies in severely immunocompromised adults because of very-low-certainty evidence and potential infectious risks [22].

CDI may mimic or precipitate an IBD flare, and diarrhea may persist after CDI treatment because of underlying disease activity. AGA recommends against conventional FMT as treatment for ulcerative colitis or Crohn disease except in clinical trials. This should not be confused with use of microbiota-based therapy for prevention of rCDI in a patient who also has IBD.

## 5. Barriers to Diagnosis and Treatment in Practice

### 5.1. Provider Uncertainty and Practice Variation

Guideline recommendations, diagnostic algorithms, and options for preventing recurrence have changed substantially. Clinicians must interpret discordant NAAT and toxin results, distinguish active infection from colonization, and understand the indications and administration requirements of newer microbiota products [18]. Institutional testing restrictions should include a method for clinicians to obtain prompt testing when fulminant CDI is suspected, particularly in patients with ileus who cannot provide an unformed stool specimen.

### 5.2. Medication Access and Payer Barriers in the United States

Cost and insurance requirements may prevent patients from receiving guideline-recommended CDI therapies. Fidaxomicin coverage may be restricted by formulary status, prior authorization, patient cost-sharing, or requirements for previous vancomycin use [9,10]. The two FDA-approved microbiota products follow different ordering and billing processes. VOS is dispensed through a specialty pharmacy and can be taken at home, whereas RBL is a clinician-administered rectal product. RBL is generally evaluated under medical-benefit pathways, including Medicare Part B for physician-administered products [46]. Coverage and payment depend on the payer, benefit, and site of administration.

Cost-effectiveness analyses suggest that microbiota-based therapies may be economically favorable in selected patients with rCDI by reducing future recurrences and downstream healthcare utilization [47,48]. Results depend on assumptions about product cost, recurrence risk, effectiveness, and subsequent healthcare utilization and do not establish affordability for individual patients.

Coverage criteria vary across payers. As of 2026, Minnesota Medicaid, for example, requires at least 3 CDI episodes within 12 months, completion of antibacterial treatment 2 to 4 days before VOS, and prescribing by or in consultation with an infectious diseases or gastroenterology specialist [49]. Product-specific ordering, storage, and administration requirements may also limit availability to selected pharmacies and treatment centers.

### 5.3. US FMT Access and Regulatory Changes

Conventional FMT remains investigational. Under its 2022 guidance, FDA exercises enforcement discretion for FMT used to treat CDI that has not responded to standard therapies when specified conditions are met. However, this policy does not apply to FMT obtained from a stool bank that collects and processes FMT product for distribution to other institutions [25] (Table 2).

OpenBiome stopped distributing investigational FMT preparations on 31 December 2024. This removed a major source of prescreened donor material [16]. Access to conventional FMT now depends more heavily on institutional donor programs operating under FDA requirements, treatment through an investigational new drug protocol, or referral to a center with an established FMT program. The loss of stool bank–derived FMT is particularly relevant to patients with severe or fulminant CDI that has not responded to antimicrobial treatment.

### 5.4. Patient Barriers and Care Transitions

Patient barriers include medication cost, transportation, caregiver support, health literacy, and preferences regarding oral or rectal administration. Prolonged vancomycin tapers may be difficult to follow. VOS requires a laxative, fasting before the first dose, and 4 capsules daily for 3 days. RBL requires administration by a healthcare professional. Patients should be counseled about recurrence symptoms and advised that repeat testing should not be used as a test of cure after symptoms resolve. Written taper calendars, caregiver involvement, and planned follow-up may improve adherence and facilitate timely evaluation of recurrent symptoms.

## 6. Practical Recommendations

### 6.1. Administration of VOS and RBL

Fecal microbiota spores, live-brpk and RBL can be prescribed by any licensed provider [46]. VOS is initiated 2 to 4 days after completing antibacterial treatment for rCDI. On the day before the first dose and at least 8 h beforehand, patients drink 296 mL of magnesium citrate and then fast for at least 8 h, except for a small amount of water. Patients take 4 capsules once daily before the first meal for 3 consecutive days. Patients with kidney disease may be unable to take magnesium citrate. In the clinical trials, such patients received 250 mL of polyethylene glycol electrolyte solution. Antibacterials should not be administered concurrently with VOS because they may interfere with the product. Subsequent antibacterial treatment should be used when clinically indicated [50].

RBL is administered as a single 150 mL rectal dose by a healthcare professional 24–72 h after the final CDI antibiotic dose. There is no need for bowel preparation, fasting, or sedation. After administration, the patient remains in the left lateral or knee–chest position for up to 15 min to reduce cramping [51]. Both therapies are generally well tolerated, with transient gastrointestinal symptoms being the most common adverse effects.

### 6.2. Coverage and Patient-Assistance Programs

Manufacturer support programs offer benefits investigation, prior-authorization assistance, specialty pharmacy coordination, and financial-assistance screening. Eligible commercially insured patients may qualify for copay assistance, whereas such programs generally exclude patients whose prescriptions are reimbursed by Medicare, Medicaid, or another government program. Uninsured or underinsured patients may qualify for separate patient-assistance programs. Eligibility and program terms should be verified at the time of prescribing because they change over time [46,52]. Because RBL must be administered 24–72 h after the last CDI antibiotic dose and VOS must be administered within 2–4 days after the final CDI antibiotic dose, coverage verification and product ordering should begin before the antibiotic course is completed (Supplementary Files S2 and S3).

### 6.3. Discharge Planning and Follow-Up

Clinicians should reassess the indication for proton-pump inhibitor therapy, avoid unnecessary systemic antibiotics, and arrange follow-up for patients at increased risk for recurrence. For patients with rCDI who will receive VOS or RBL, coverage verification and product ordering should begin before the antibiotic course is completed. Referral for conventional FMT should also be initiated early when indicated. Oral vancomycin prophylaxis may be considered for selected patients at high risk for recurrence who require subsequent systemic antibiotics, but evidence is limited, guideline recommendations differ, and routine prophylaxis is not recommended.

Common barriers and potential responses are summarized in Table 3.

## 7. Future Directions

Important evidence gaps remain. VOS, RBL, and conventional FMT have not been compared directly in randomized trials. Validated methods are needed to estimate recurrence risk, and evidence regarding the safety and effectiveness of microbiota-based therapies in severely immunocompromised patients remains limited. Biomarkers of microbiota engraftment also require validation before clinical use. Ibezapolstat, a DNA polymerase IIIC inhibitor, remains investigational after phase 2 evaluation [53]. Future studies should report time to treatment, insurance approval, patient out-of-pocket costs, treatment completion, and outcomes according to insurance type and geographic setting.

## 8. Conclusions

In the United States, successful CDI management requires accurate diagnosis, appropriate antibiotic selection, assessment of recurrence risk, and early attention to coverage, product availability, regulatory pathways, and care transitions. NAAT and toxin results should be interpreted in conjunction with symptoms and alternative causes of diarrhea. Several guidelines prefer fidaxomicin for initial nonfulminant CDI, although oral vancomycin remains an effective alternative when fidaxomicin is unavailable. For rCDI, conventional FMT, VOS, and RBL provide recurrence prevention options but differ in route of administration, timing, eligibility, and availability. The U.S. discontinuation of bezlotoxumab and reduced access to stool bank-derived FMT have further limited available options. When microbiota-based treatment is indicated, coverage and product availability should be assessed before the antibiotic course ends.

## Figures and Tables

**Table 1:** Pivotal Trials of FDA-Approved Microbiota Products for Prevention of Recurrent CDI.

Trial/Product	Population	Prior Episodes/Entry Criteria	Prespecified Primary Endpoint	Effect Estimate	Follow-Up
ECOSPOR III/VOS	182 adults with multiply recurrent CDI after symptom resolution with standard-of-care antibiotics; toxin-positive entry and recurrence confirmation.	At least 3 CDI episodes within 12 months, including the qualifying episode.	Recurrent toxin-positive CDI requiring treatment through week 8.	12.4% vs. 39.8%; relative risk 0.32 (95% CI, 0.18–0.58) [13].	Primary endpoint at 8 weeks; follow-up through 24 weeks.
PUNCH CD3/RBL	Adults with recurrent CDI after standard-of-care antibiotics; 289 randomized and 267 treated.	At least 1 recurrence after a primary episode, or at least 2 severe episodes requiring hospitalization within the preceding year.	Treatment success, defined as absence of CDI diarrhea within 8 weeks.	Bayesian estimate 70.6% vs. 57.5%; difference 13.1 percentage points (95% CrI, 2.3–24.0); posterior probability of superiority 0.991 [12].	Primary endpoint at 8 weeks; follow-up through 6 months.

**Table 2:** Recent Changes in Adult CDI Management.

Change	Practical Implication
IDSA/SHEA and ESCMID prefer fidaxomicin for initial nonfulminant CDI when available [19,21]	Oral vancomycin remains an effective guideline-supported alternative when fidaxomicin is unavailable or not covered [19,21].
Merck discontinued Zinplava in the United States in January 2025 [17]	Bezlotoxumab is no longer available in U.S. practice. Other options include antibiotic strategies, conventional FMT, and FDA-approved microbiota products, as clinically appropriate [17].
RBL approved in 2022 and VOS in 2023 [12,13]	Both products prevent rCDI but differ in route, administration, timing, and access requirements [46,50,51].
FDA issued final FMT enforcement-discretion guidance in 2022 [25]	Enforcement discretion does not extend to FMT obtained from a stool bank that distributes material to other institutions [25]
OpenBiome stopped distributing investigational FMT preparations in December 2024 [16]	Conventional FMT availability now depends on institutional donor programs, applicable FDA requirements, IND protocols, or referral to experienced centers [16,25]

Abbreviations: CDI, *Clostridioides difficile* infection; ESCMID, European Society of Clinical Microbiology and Infectious Diseases; FDA, US Food and Drug Administration; FMT, fecal microbiota transplantation; IDSA, Infectious Diseases Society of America; IND, investigational new drug; rCDI, recurrent CDI; SHEA, Society for Healthcare Epidemiology of America.

**Table 3:** Practical Barriers and Potential Responses in CDI Care.

Domain	Barrier	Potential Response
Diagnostic stewardship	Low pre-test probability testing, Performing test of cure, NAAT-positive and EIA-negative cases	Test only symptomatic patients with ≥3 unformed stools/24 h; avoid test-of-cure; use multistep algorithms when available; interpret discordant results clinically [4,28,29,30,31,32,33,34]
Treatment access	Fidaxomicin, VOS, or RBL limited by cost, formulary restrictions, or prior authorization	Involve pharmacy/case management early; use PA templates; start access planning during CDI treatment [9,10,49,52].
Recurrence prevention	Delays obtaining fecal microbiota therapy after antibiotics	Identify eligible patients early; coordinate VOS shipment, RBL administration, or specialty referral before antibiotic completion [12,13,22,46,50,51].
FMT availability	Limited access to conventional FMT outside specialized centers	Confirm institutional or IND availability; refer when unavailable locally [16,25]
Care transitions	Confusion about tapers, recurrence symptoms, or follow-up	Provide written instructions, taper calendars, recurrence warnings, and arrange early outpatient follow-up [24,50,51]

Abbreviations: CDI, *Clostridioides difficile* infection; FMT, fecal microbiota transplantation; IND, investigational new drug; NAAT, nucleic acid amplification test; PA: Prior authorization.

## Data Availability

No new data were created or analyzed in this study. Data sharing is not applicable to this article.

## Supplementary Materials

The following supporting information can be downloaded at: https://www.mdpi.com/article/10.3390/antibiotics15101004/s1, Supplementary File S1, Fidaxomicin Prior Authorization Letter Template; Supplementary File S2, VOS Prior Authorization Letter Template; Supplementary File S3, RBL Prior Authorization Letter Template. The references [54,55,56] are cited in the Supplementary Materials.

## Abbreviations

The following abbreviations are used in this manuscript:

Abbreviation	Definition
ACG	American College of Gastroenterology
AGA	American Gastroenterological Association
CDI	*Clostridioides difficile* infection
EIA	Enzyme immunoassay
ESCMID	European Society of Clinical Microbiology and Infectious Diseases
FDA	U.S. Food and Drug Administration
FMT	Fecal microbiota transplantation
GDH	Glutamate dehydrogenase
IBD	Inflammatory bowel disease
IBS	Irritable bowel syndrome
IDSA/SHEA	Infectious Diseases Society of America and Society for Healthcare Epidemiology of America
IND	Investigational new drug
NAAT	Nucleic acid amplification test
PolC	DNA polymerase IIIC
rCDI	Recurrent *Clostridioides difficile* infection
RBL	Fecal microbiota, live-jslm
SoC	Standard of care
VOS	Fecal microbiota spores, live-brpk
