# Supplementary material for: Barriers to Diagnosis and Treatment of Clostridioides difficile Infection: A Narrative Review
Source: Antibiotics (Basel). 2026 Oct 10;15(10):1004. doi: 10.3390/antibiotics15101004 (PMC13700867; doi:10.3390/antibiotics15101004)
Supplement: Supplementary file 1 [file antibiotics-15-01004-s001.zip › antibiotics-4556122-supplementary.pdf]

Supplementary Materials

Review
Barriers to Diagnosis and Treatment of *Clostridioides difficile* Infection: A Narrative Review

Parul Berry [1], Vishwesh Bharadiya [1] and Sahil Khanna [2,*]

[1] Department of Internal Medicine, Mayo Clinic, Rochester, MN 55905, USA
[2] Division of Gastroenterology and Hepatology, Mayo Clinic, Rochester, MN 55905, USA
* Correspondence: khanna.sahil@mayo.edu

Prior Authorization Letter Templates for Therapies Used in U.S. *Clostridioides difficile* Infection Care

Contents

- Supplementary File S1. Fidaxomicin Prior Authorization Letter Template
- Supplementary File S2. VOWST Prior Authorization Letter Template
- Supplementary File S3. REBYOTA Prior Authorization Letter Template

Abbreviations: CDI, *Clostridioides difficile* infection; PA, prior authorization; RBL, fecal microbiota, live-jslm; VOS, fecal microbiota spores, live-brpk.

Scope note: These templates are designed for U.S. clinical and payer settings. Clinicians should replace all bracketed fields with patient-specific information, verify the requested regimen against current prescribing information, and adapt each letter to the applicable payer and institutional requirements.

Supplementary File S1. Fidaxomicin Prior Authorization Letter Template

Note to users: This U.S. focused template is intended as administrative support for licensed clinicians. Replace bracketed fields with patient-specific details, confirm the requested regimen against the current prescribing information, and adapt the letter to payer requirements and local institutional policy.

Fidaxomicin (DIFICID) Prior Authorization Letter

**Field**	**Patient-specific entry**
**Patient**	______________________ DOB: ______________________
**Insurance ID**	______________________ Date: ______________________
**Diagnosis**	Initial or nonrecurrent CDI, ICD-10-CM A04.72; or recurrent CDI, ICD-10-CM A04.71, as applicable.
**Medication requested**	Fidaxomicin (DIFICID)
**Clinical history**	[X CDI episodes/recurrences; prior therapies; dates]
**Risk factors**	[age, immunosuppression, IBD, recent antibiotics, hospitalization, etc.]
**Requested regimen**	Fidaxomicin 200 mg orally twice daily for 10 days, or fidaxomicin 200 mg twice daily followed by 200 mg once every other day from day 7 through day 25 as an extended-pulsed regimen if clinically appropriate.
**Attachments**	See checklist below

To Whom It May Concern,

I am requesting coverage for fidaxomicin for my patient with *Clostridioides difficile* infection (CDI). The patient has experienced [initial CDI / first recurrence / multiple recurrences] and is at increased risk for further recurrence or complications because of [age >=65 years, immunocompromised state, inflammatory bowel disease, recent hospitalization, recent systemic antibiotics, severe CDI, prior recurrence, or other patient-specific risk factors].

The 2021 IDSA/SHEA focused update suggests fidaxomicin rather than a standard course of vancomycin for both initial and recurrent CDI when feasible because fidaxomicin provides similar initial clinical cure with lower recurrence risk. Vancomycin remains an acceptable alternative; however, this patient's recurrence risk, recurrence-related adverse outcomes, and clinical context support fidaxomicin as the guideline-supported option.

Preventing recurrence is clinically important because recurrent CDI is associated with repeated antibiotic exposure, emergency department visits, hospitalizations, morbidity, and substantial healthcare costs. Published cost syntheses estimate recurrent CDI-associated annual per-patient direct medical costs in the range of approximately $67,000 to $82,000.

Coverage of fidaxomicin is medically necessary and evidence-based for this patient. Approval would align with national guideline recommendations and may reduce the likelihood of further CDI recurrence, avoidable healthcare utilization, and additional antibiotic exposure.

Requested regimen:

- Fidaxomicin 200 mg orally twice daily for 10 days, or fidaxomicin 200 mg twice daily followed by 200 mg once every other day from day 7 through day 25 as an extended-pulsed regimen if clinically appropriate.

Suggested documentation to attach:

- Recent clinical note documenting CDI symptoms and recurrence history
- C. difficile test results and dates
- Prior CDI treatment history, including vancomycin, metronidazole, or fidaxomicin exposure
- Hospitalization or emergency department records, if applicable
- Risk-factor documentation, such as immunosuppression, IBD, age >=65 years, renal disease, or recent systemic antibiotic exposure

Thank you for your timely review of this medically necessary request.

Sincerely,

Provider Name / Signature: ________________________________

NPI: ____________________ Phone/Fax: ____________________

Selected supporting references

1. Johnson S, Lavergne V, Skinner AM, et al. Clinical Practice Guideline by IDSA/SHEA: 2021 Focused Update Guidelines on Management of Clostridioides difficile Infection in Adults. Clin Infect Dis. 2021;73(5):e1029-e1044.
2. Louie TJ, Miller MA, Mullane KM, et al. Fidaxomicin versus vancomycin for Clostridium difficile infection. N Engl J Med. 2011;364(5):422-431.
3. Crook DW, Walker AS, Kean Y, et al. Fidaxomicin versus vancomycin for Clostridium difficile infection: meta-analysis of pivotal randomized controlled trials. Clin Infect Dis. 2012;55 Suppl 2:S93-S103.
4. Reveles KR, Yang M, Garcia-Horton V, Edwards ML, Guo A, Lodise T, et al. Economic impact of recurrent Clostridioides difficile infection in the USA: a systematic literature review and cost synthesis. Adv Ther. 2023;40(7):3104-3134.

Supplementary File S2. VOS Prior Authorization Letter Template

Note to users: This template is intended as administrative support for licensed clinicians. Replace bracketed fields with patient-specific details, confirm the requested regimen against the current prescribing information, and adapt the letter to payer requirements and local institutional policy.

VOWST (fecal microbiota spores, live-brpk) Prior Authorization Letter

**Field**	**Patient-specific entry**
**Patient**	______________________ DOB: ______________________
**Insurance ID**	______________________ Date: ______________________
**Diagnosis**	Recurrent Clostridioides difficile infection (rCDI) (ICD-10 A04.71)
**Medication requested**	VOWST (fecal microbiota spores, live-brpk)
**Clinical history**	[X CDI episodes/recurrences; prior therapies; dates]
**Risk factors**	[age, immunosuppression, IBD, recent antibiotics, hospitalization, etc.]
**Requested regimen**	VOWST: 4 capsules orally once daily for 3 consecutive days, initiated 2 to 4 days after completion of antibacterial treatment for recurrent CDI, with bowel preparation per prescribing information and clinical judgment.
**Attachments**	See checklist below

To Whom It May Concern,

I am requesting coverage for VOWST (fecal microbiota spores, live-brpk), an FDA-approved orally administered microbiota-based therapy, for prevention of recurrent *Clostridioides difficile* infection (CDI) in my adult patient following completion of antibacterial treatment for recurrent CDI.

This patient has experienced [X recurrences] despite appropriate CDI-directed antibiotic therapy and remains at elevated risk for further recurrence because of [age >=65 years, immunocompromised state, inflammatory bowel disease, recent hospitalization, recent systemic antibiotics, severe CDI, multiple prior CDI episodes, or other patient-specific risk factors]. The requested therapy is not being used to treat active CDI; it is requested to prevent further recurrence after completion of antibacterial treatment, consistent with the FDA-approved indication.

Clinical evidence supports VOWST for this indication. In the phase 3 ECOSPOR III trial, SER-109/VOWST significantly reduced CDI recurrence through 8 weeks compared with placebo (12.4% vs 39.8%). The AGA clinical practice guideline supports select use of fecal microbiota-based therapies after standard-of-care antibiotics in appropriate adults with recurrent CDI.

Access to conventional donor-derived fecal microbiota transplantation varies by institution and may be limited where local donor programs or referral pathways are unavailable. VOWST provides a standardized, FDA-regulated option for microbiome restoration in a patient with ongoing recurrence risk. Preventing recurrence may reduce avoidable healthcare utilization, repeated antibiotic exposure, hospitalization risk, and patient morbidity.

Approval of VOWST is medically necessary and consistent with the patient's recurrent CDI history, risk profile, and available guideline-supported treatment pathways.

Requested regimen:

- VOWST: 4 capsules orally once daily for 3 consecutive days, initiated 2 to 4 days after completion of antibacterial treatment for recurrent CDI, with bowel preparation per prescribing information and clinical judgment.

Suggested documentation to attach:

- Clinical notes documenting recurrent CDI episodes
- C. difficile test results and dates
- Documentation of completed or planned antibacterial treatment for recurrent CDI
- Prior CDI treatment history
- Risk-factor documentation
- Medication list and relevant contraindication/allergy review

Thank you for your timely review of this medically necessary request.

Sincerely,

Provider Name / Signature: ________________________________
NPI: ____________________ Phone/Fax: ____________________

Selected supporting references

1. U.S. Food and Drug Administration. VOWST. U.S. Food and Drug Administration; 2023. Available from: https://www.fda.gov/vaccines-blood-biologics/vowst. Accessed June 9, 2026.
2. Feuerstadt P, Louie TJ, Lashner B, et al. SER-109, an oral microbiome therapy for recurrent Clostridioides difficile infection. N Engl J Med. 2022;386(3):220-229.
3. Peery AF, Kelly CR, Kao D, Vaughn BP, Lebwohl B, Singh S, et al. AGA Clinical Practice Guideline on Fecal Microbiota-Based Therapies for Select Gastrointestinal Diseases. Gastroenterology. 2024;166(3):409-434.
4. Reveles KR, Yang M, Garcia-Horton V, Edwards ML, Guo A, Lodise T, et al. Economic impact of recurrent Clostridioides difficile infection in the USA: a systematic literature review and cost synthesis. Adv Ther. 2023;40(7):3104-3134.

Supplementary File S3. RBL Prior Authorization Letter Template

Note to users: This template is intended as administrative support for licensed clinicians. Replace bracketed fields with patient-specific details, confirm the requested regimen against the current prescribing information, and adapt the letter to payer requirements and local institutional policy.

REBYOTA (fecal microbiota, live-jslm) Prior Authorization Letter

**Field**	**Patient-specific entry**
**Patient**	______________________ DOB: ______________________
**Insurance ID**	______________________ Date: ______________________
**Diagnosis**	Recurrent Clostridioides difficile infection (rCDI) (ICD-10 A04.71)
**Medication requested**	REBYOTA (fecal microbiota, live-jslm)
**Clinical history**	[X CDI episodes/recurrences; prior therapies; dates]
**Risk factors**	[age, immunosuppression, IBD, recent antibiotics, hospitalization, etc.]
**Requested regimen**	REBYOTA: single 150 mL rectal dose administered 24 to 72 hours after the last dose of antibiotics for CDI, per prescribing information.
**Attachments**	See checklist below

To Whom It May Concern,

I am requesting coverage for REBYOTA (fecal microbiota, live-jslm), an FDA-approved microbiota-based therapy, for prevention of recurrent *Clostridioides difficile* infection (CDI) in my adult patient following completion of antibiotic treatment for recurrent CDI.

This patient has experienced [X recurrences] despite standard-of-care CDI-directed antibiotic therapy and remains at elevated risk for further recurrence because of [age >=65 years, immunocompromised state, inflammatory bowel disease, recent hospitalization, recent systemic antibiotics, severe CDI, multiple prior CDI episodes, or other patient-specific risk factors]. The requested therapy is not being used to treat active CDI; it is requested to prevent recurrence after completion of antibiotic treatment, consistent with the FDA-approved indication.

Clinical evidence supports REBYOTA for this indication. In the efficacy analysis supporting approval, treatment success at 8 weeks was higher with REBYOTA than placebo (70.6% vs 57.5%). The AGA clinical practice guideline supports select use of fecal microbiota-based therapies after standard-of-care antibiotics in appropriate adults with recurrent CDI.

Access to conventional donor-derived fecal microbiota transplantation varies by institution and may be limited where local donor programs or referral pathways are unavailable. REBYOTA provides a standardized, FDA-regulated option for microbiome restoration after antibiotic treatment in a patient with ongoing recurrence risk. Preventing recurrence may reduce avoidable healthcare utilization, repeated antibiotic exposure, hospitalization risk, and patient morbidity.

Approval of REBYOTA is medically necessary and consistent with the patient's recurrent CDI history, risk profile, and available guideline-supported treatment pathways.

Requested regimen:

- REBYOTA: single 150 mL rectal dose administered 24 to 72 hours after the last dose of antibiotics for CDI, per prescribing information.

Suggested documentation to attach:

- Clinical notes documenting recurrent CDI episodes
- C. difficile test results and dates
- Documentation of completed or planned antibiotic treatment for recurrent CDI
- Prior CDI treatment history
- Risk-factor documentation
- Medication list and review for relevant allergies or contraindications

Thank you for your timely review of this medically necessary request.

Sincerely,

Provider Name / Signature: ________________________________
NPI: ____________________ Phone/Fax: ____________________

Selected supporting references

1. U.S. Food and Drug Administration. REBYOTA. U.S. Food and Drug Administration; 2023. Available from: www.fda.gov/vaccines-blood-biologics/vaccines/rebyota. Accessed June 9, 2026.
2. Khanna S, Assi M, Lee C, Yoho D, Louie T, Knapple W, et al. Efficacy and safety of RBX2660 in PUNCH CD3, a phase III randomized, double-blind, placebo-controlled trial for prevention of recurrent Clostridioides difficile infection. Drugs. 2022;82(15):1527-1538.
3. Peery AF, Kelly CR, Kao D, Vaughn BP, Lebwohl B, Singh S, et al. AGA Clinical Practice Guideline on Fecal Microbiota-Based Therapies for Select Gastrointestinal Diseases. Gastroenterology. 2024;166(3):409-434.
4. Reveles KR, Yang M, Garcia-Horton V, Edwards ML, Guo A, Lodise T, et al. Economic impact of recurrent Clostridioides difficile infection in the USA: a systematic literature review and cost synthesis. Adv Ther. 2023;40(7):3104-3134.
